# Contrasting Foraging Patterns: Testing Resource-Concentration and Dilution Effects with Pollinators and Seed Predators

**DOI:** 10.3390/insects7020023

**Published:** 2016-06-03

**Authors:** Alexandria Wenninger, Tania N. Kim, Brian J. Spiesman, Claudio Gratton

**Affiliations:** 1 Great Lakes Bioenergy Research Center and Department of Entomology, University of Wisconsin-Madison, Madison, WI 53706, USA; akwenninger@alaska.edu (A.W.); bspiesman@wisc.edu (B.J.S.); cgratton@wisc.edu (C.G.); 2 Department of Biology and Wildlife, University of Alaska Fairbanks, Fairbanks, AK 99775, USA

**Keywords:** optimal foraging, scale, prairie, pollination, seed predation, Apidae, Formicidae

## Abstract

Resource concentration effects occur when high resource density patches attract and support more foragers than low density patches. In contrast, resource dilution effects can occur if high density patches support fewer consumers. In this study, we examined the foraging rates of pollinators and seed predators on two perennial plant species (*Rudbeckia triloba* and *Verbena stricta*) as functions of resource density. Specifically, we examined whether resource-dense patches (densities of flower and seeds on individual plants) resulted in greater visitation and seed removal rates, respectively. We also examined whether foraging rates were context-dependent by conducting the study in two sites that varied in resource densities. For pollinators, we found negative relationships between the density of flowers per plant and visitation rates, suggesting dilution effects. For seed predators, we found positive relationships consistent with concentration effects. Saturation effects and differences in foraging behaviors might explain the opposite relationships; most of the seed predators were ants (recruitment-based foragers), and pollinators were mostly solitary foragers. We also found that foraging rates were site-dependent, possibly due to site-level differences in resource abundance and consumer densities. These results suggest that these two plant species may benefit from producing as many flowers as possible, given high levels of pollination and low seed predation.

## 1. Introduction

Theory suggests that consumers should forage in ways that will maximize the benefits received from resources, while minimizing the energetic costs of obtaining those resources [1]. One way to accomplish this is to forage in areas with concentrated resources as these resource-dense patches should be easier to find and provide more resources to distribute foraging efforts (resource concentration effects (RCE) [2,3]). Additionally, because of the greater density of resources, foragers can minimize the energetic costs of moving between patches to find food [1]. Many studies have looked for the existence of RCE in a variety of different consumer-resource systems with predators [4], herbivores [5,6,7], pollinators [8,9] and seed predators [10]. While theory suggests that foragers should distribute their foraging efforts in ways to maximize fitness [11], results from empirical studies are mixed. For instance, Otway *et al.* [6] found a negative relationship between resource density and insect load (a resource dilution effect) for nine specialist herbivores, while Tubbesing *et al.* [9] found resource concentration effects for bumble bees.

One reason for the inconsistent results and contrasting patterns could be due to differences in the search modes of the consumers, which could vary with spatial scales. Consumers use a variety of different cues (visual, contact, and olfactory) to inform foraging decisions and employ different strategies of obtaining resources efficiently. Because decision-making processes are often times hierarchical [6,12,13,14] where foragers must first select a patch in which to forage (between-patch selection) and then target resources once inside a patch (within-patch selection [3,15,16]), relationships between resource density and foraging rates might vary with spatial scale. For example, Jha and Vandermeer [13] observed positive relationships between visitation rates of bees and coffee floral resources at large (100 m) spatial scales (resource concentration effects), but found negative relationships at small (plant-level) spatial scales (resource dilution effects). Resource-dense patches may have been easier to find at large spatial scales, especially for foragers that use visual cues. However, once inside a patch, other cues (contact or olfactory) may have been used to assess resource quality and selection [12,17,18,19]; each cue having varying relationships with resource density. Furthermore, within a given spatial scale, life-history differences of consumers might influence foraging rates and resource density relationships. For example, social animals that convey information to others (e.g., mass recruitment behavior, such as leaving pheromone trails, or signaler limited recruitment, such as the waggle dance [20]) might exhibit a resource concentration effect, in efforts to increase foraging efficiency and colony performance [21,22]. In contrast, the opposite effect (a resource dilution effect), or no density response may also be observed for solitary foragers, if they are avoiding competition [14], or if high resource density patches provide fewer resources for foragers to distribute their efforts (*i.e.*, saturation effects [19,23]).

From the plant’s perspective, different relationships between resource densities and foraging rates could have varying consequences for fitness. If plants with greater floral resources attract more pollinators per unit resource (and thus, have greater pollination rates and seed set), then producing many flowers might be the optimal strategy for plants. On the other hand, if plants with a greater number of seeds (due to increased pollination) attract seed consumers (thus increasing seed predation), then producing many floral resources might not be the best strategy. Therefore, insect-pollinated plants may face tradeoffs between investing energetic resources towards traits, such as size and flowers that will attract mutualistic partners, while avoiding antagonistic interactions (e.g., seed predation and herbivory). If antagonistic and mutualistic partners use the same plant traits to select host plants [24], there might be opposing selection pressures for these plant traits, resulting in an optimal plant phenotype.

In this study, we experimentally examined the foraging patterns of pollinators and seed predators across a range of resource densities. Specifically, we asked how resource densities (number of flowers and seeds per plant) affected visitation and seed removal rates, respectively. We predicted that if resource concentration effects occur, the per-unit resource visitation and seed removal rates would be greater on resource-dense plants compared to resource-sparse plants, because resource-dense plants could provide a more cost-efficient foraging opportunity [2]. Alternatively, if the per-unit visitation and seed removal rates are low in resource-dense plants, it would suggest resource dilution effects [6]. Because pollinators and seed predators have been known to respond to resource variation at both small (plant-level) and large (field-level) scales [12,13,14,25], we examined whether foraging rate and resource density relationships were site-dependent. We predicted that in a site with low naturally-occurring resources, the relationships between resource density and foraging rates would be weak because consumers would flock to plants at all resources levels equally compared to the site where there are high levels of naturally-occurring resources available. Finally, we were interested in the fitness consequences of concentrated floral resources for the plants by examining the relationships between resource density and the final number of seeds remaining after predation.

## 2. Experimental Section

### 2.1. Study Organisms and Plant Preparation

Our focal species consisted of two prairie plants, *Rudbeckia triloba* L. (Brown-eyed Susan) and *Verbena stricta* (Hoary Vervain), both native to Wisconsin (USA). *Verbena stricta* is a perennial, outcrossing species with low seed set through self-fertilization [26]. *Rudbeckia triloba*, on the other hand, can range from biennial to weak perennial and can self-pollinate, though members of the genus *Rudbeckia* do benefit from outcrossed pollination by insects [27]. In the upper mid-west region of the USA, both plant species produce flowers continuously during the months of July through September. Neither *R. triloba* nor *V. stricta* rely on special mechanisms of seed dispersal; seeds fall out of the seed heads as they dry and do not contain elaiosomes to facilitate ant dispersal. Both species have similar seed sizes (*R. triloba* = 1.3 ± 0.49 (mean ± SE) mg; *V. stricta* = 1.5 ± 0.35 (mean ± SE) mg). *Verbena stricta* and *R. triloba* were obtained from a nursery (Prairie Nursery, Westfield, WI, USA) in July 2013 and were kept in a greenhouse for about two weeks at 22.2 °C (21.5 °C–23.0 °C) until flowers opened.

Once flowers opened, we separated plants into three plant-level resource density treatments (“low”, “medium” and “high” floral resource densities, hereafter “flower treatments”). For *V. stricta*, individual plants in the low flower treatment had <20 flowers (mean = 12.81 flowers, range = 6–19); medium flower treatment had >20 flowers on a single stem (mean = 24.43 flowers, range = 20–35); and high flower treatment had multiple stems with flowers (mean = 39.06 flowers, range =18–67). For *R. triloba*, individuals in the low flower treatment had 1–2 flower heads; medium flower treatment had 4–5 flower heads; and high flower treatment had >7 flower heads. For *R. triloba*, we snipped off flowers for the low treatment category; flowers did not need to be cut for any other treatments. We measured the height of each individual plant prior to deployment in the field.

### 2.2. Study Sites

The experiment took place in two prairie fields in Wisconsin (Dane County, WI, USA) from 31 July–6 October 2013. Previous work in our lab shows that one study site (Harvey’s Marsh Waterfowl Production Area, hereafter “Harvey’s Marsh”) is a low resource site with relatively low floral cover (0.21 individuals in flower m^−1^ quadrat, [28]) compared to the other site (Brooklyn Wildlife Natural Area, hereafter “Brooklyn”), which is a high resource site with higher floral cover (1.48 individuals in flower m^−1^ quadrat, [28]). Although *V. stricta* and *R. triloba* were not present at either sites, they are present in other similar grasslands throughout the region [28]. Sites varied in sizes (Brooklyn: 14.28 km^2^, Harvey’s Marsh: 2.5 km^2^), but we confined our sampling efforts to a 50 m × 100 m area.

Previous work in our lab has also shown that these sites varied in the composition of the pollinator [28] and seed predator (mostly ants) communities [29]. In August 2013, pan traps captured a greater number of individual bees (8.2 individuals per trap) at Brooklyn (a high resource site) compared to 5.8 individuals per trap at Harvey’s Marsh (a low resource site). At Brooklyn, the pollinator species richness of bees (Hymenoptera: Apocrita), flies (Diptera), beetles (Coleoptera) and butterflies (Lepidoptera) were 35, 10, 5 and 3, species respectively. In contrast, there was a lower number of species at Harvey’s Marsh (26, 6, 3 and 1 species of bee, flies, beetles and butterflies, respectively). Pitfall trapping at these sites also revealed that ground-dwelling arthropod abundance at Brooklyn was greater compared to Harvey’s Marsh. At Brooklyn, a total of 3141 arthropods were collected from pitfall samples. Ants were the most dominant taxon (83.1% of the individuals, mostly *Lasius neoniger*) followed by crickets (13.9%) and carabid beetles (3.2%). At Harvey’s Marsh, 1156 individuals were captured, with ants comprising the greatest number of individuals (63.9%), followed by spiders (33.7%) and crickets (1.8%).

### 2.3. Pollination Field Experiment

In August, experimental plants were placed along three 100-m transects, each transect separated by 25 m. On each transect, potted plants were placed at stations every 20 m (15 stations in total per site; Figure S1). At each of the 15 stations, we placed one randomly-selected *R. triloba* and *V. stricta* from one of the three plant-level flower treatments (low, medium, high). Plants at each station were separated by a distance of 1.5 m. While wild, flowering plants were in the vicinity of the experimental plants, they were at least 2 m away from plants at each station. To account for seed set due to non-insect pollination (*i.e.*, abiotic and selfing), we placed additional plants that were covered with a fine mesh (2 mm in size) to prevent insect pollination at nine of the 15 stations (hereafter “control” plants). Three individuals from each floral resource category were placed out into the field as control plants. Therefore, at each of the two sites, we had 15 plants “open” to pollination (five individuals of each flower category) and nine “control” plants (three individuals of each flower category), for each of the two plant species (*n* = 96 individuals overall).

All plants were placed in the field for only one week (July 30–August 6) because we were interested in the short-term attractiveness of plants to pollinators. Plants were kept in their pots to avoid transplant shock if buried directly into the ground. Plants were held upright with bamboo stakes and landscape staples and watered twice in the field; once at the start of the experiment and once mid-week. We performed two-timed pollinator observations on each experimental plant during the week (August 1 and August 6). The two observation days were similar in weather: sunny (>20 °C) with little wind (<3 m/s); and on both days, observations occurred in the late morning/early afternoon. Each observation per plant lasted 15 min, during which the observer was seated about 1.25 m from the plants. We recorded the number of visits (each new approach with contact was considered a visit), visitation time (time on plant in seconds) and identified visitors to the lowest possible taxonomic level. At the end of the week, all plants were covered with a mesh bag to prevent further pollination and brought back to the greenhouse. Because both plant species produced flowers continuously over the growing season, the number of flowers at the start of the experiment might differ from the number of flowers at the end of the visitation experiment. Therefore, we counted the number of open flowers upon return to the lab to ensure that plants still remained in their original flower density treatments. Plants were sprayed with a contact insecticide to remove any arthropods and maintained in the greenhouse until seed set. Once seed set had occurred, we counted all seeds from each plant.

### 2.4. Seed Predation Experiment

All seeds produced from individuals in the pollination experiment were collected separately and counted before returning them to the field to estimate seed removal rates. Because we were interested in the fitness consequences of concentrated resources for each plant, seeds were returned to the exact same locations in the field as the pollination experiment to link individual plant results from the two experiments. Seeds were placed out on 10-cm petri dishes covered with 2.5 cm wire mesh to deter mammals from feeding. Seeds were left out in the field for a period of 48 h from 7–9 October 2013, which coincided with the seed presence of other naturally-occurring plants in the field. The dominant insect seed predator at these sites was the ant species *Lasius* *neoniger*. These ants are not effective seed dispersers [30], but rather predators and scavengers and will collect fallen seeds to sustain developing brood. Upon return from the field, all intact seeds remaining were counted. Seeds were only counted as “intact” if they were whole or contained the majority of their endosperm.

### 2.5. Statistical Analysis

Foraging rates were calculated in two ways; total and per-unit resource. For example, the total pollination rate was determined as the total number of visits to each plant, and the per-unit resource pollination rate was the total number of visits per plant divided by the mean number of flowers on the plant. We also quantified the total visitation time on a plant as the sum of the time that each visitor stayed on the plant during both of the 15-min observation periods. The total visitation time was able to exceed the 30-min total observation period because multiple pollinators could visit the plant simultaneously. For seed removal rates, we determined the total number of seeds removed and the per-unit resource seed removal rates (the number of seeds removed and divided by the initial number of seeds). To determine how the amount of floral resources influenced pollination rates (total and per-unit resource), total visitation time, seed removal rates (total and per-unit resource) and the final number of seeds remaining at the end of the experiment (after pollination and seed removal), we preformed separate general linear models (GLM, α = 0.05) on each of these response variables with flower treatments (low, medium, high resources) as a fixed effect. We treated flower treatment as categorical rather than continuous to avoid artefactual covariance by having the number of flowers both as predictor and part of the response variables (in the cases of per-unit resource visitation rates). We were also interested site-level effects on these response variables. Because there were only two sites, we included site as a fixed factor and tested interactions between site and flower treatments [31]. Plant height and the number of flowers on heterospecific plants at each station could also influence visitation and seed removal rates; therefore, we examined whether including these covariates in the model would increase model fit. However, these covariates were not significant, and models with these extra variables did not improve model fit (ΔAIC = 1.9 to 4.3). Therefore, we did not include these covariates in the final model. For all analyses, we assumed Gaussian distributions and checked residuals to determine whether they were normally and homogenously distributed. If necessary, we log-transformed or square-root transformed the data. All analyses were performed in R 3.1.0 [32].

## 3. Results

### 3.1. Flower Visitation

**Figure 1:**
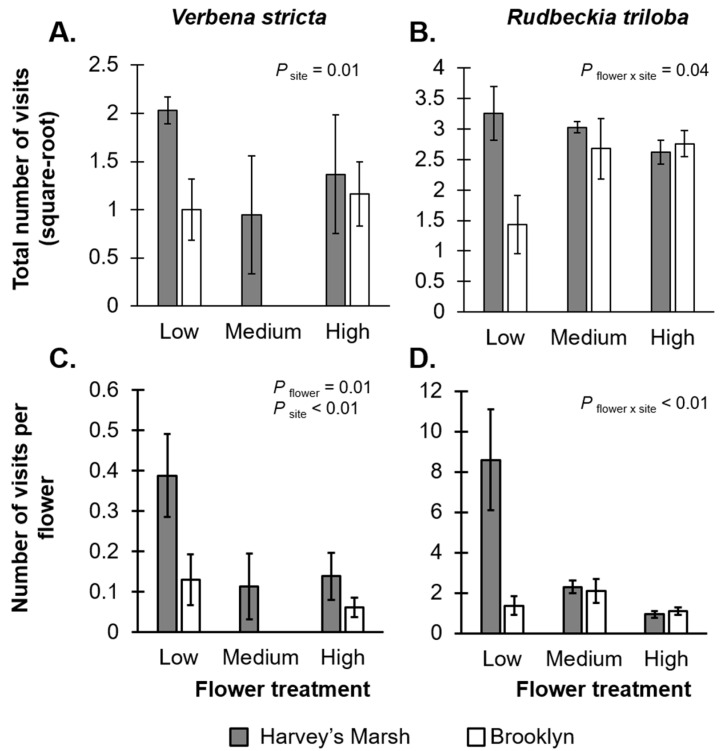
Flower treatment and site comparisons of *Verbena stricta* (left side) and *Rudbeckia triloba* (right side) on the total number of pollinator visits (**A**,**B**) and the number of visits per floral resource (**C**,**D**) within 30 min of observation. Grey bars represent rates at Harvey’s Marsh (a low resource density site); white bars represent rates at Brooklyn (a high resource density site). Error bars represent ±1 SE. The total number of pollinator visits was square-root transformed. Note: no visits occurred on *V. stricta* plants with a medium number of flowers at the high density site.

We recorded 236 visits to *R. tribola* and 75 visits to *V. stricta.* The most commonly-observed pollinator insects visiting both *V. stricta* and *R. triloba* was syrphid flies (mainly *Toxomerus marginatus*, 34.4%–83.3% of visits) and halictid bees (mainly *Augochlora pura*, 14.8%–30.6% of visits) at both sites. *Verbena* *stricta* was also visited by *Apis mellifera* (18.6% visits). For *V. stricta*, there was a marginally significant, negative relationship between flower density treatment and the total number of visits (*F*_2,24_ = 2.67, *p* = 0.08; Figure 1A, Table S1A) and a significant, negative relationship with the per-unit resource visitation rate (*F*_2,24_ = 5.42, *p* = 0.01; Figure 1C, Table S1A), suggesting resource dilution effects. There was also a significant site effect on visitation rates (total visits: *F*_1,24_ = 7.77, *p* = 0.01; visits per unit-resource: *F*_1,24_ = 8.02, *p* < 0.01); there were more visits to plants at the Harvey’s Marsh (a low resource site) than at Brooklyn (a high resource site). There was no interaction between flower treatment and site on visitation rates (total visits: *F*_2,24_ = 0.05, *p* = 0.94; visits per unit-resource: *F*_2,24_ = 1.08, *p* = 0.35) or the total time on plants (*F*_2,24_ = 1.45, *p* = 0.25; Table S1A).

For *R. triloba*, there was an interaction between site and flower treatment on both the total visitation rate (*F*_2,24_ = 3.65, *p* = 0.04; Figure 1B, Table S1B) and per-unit resource visitation rates (*F*_2,24_ = 6.67, *p* < 0.01; Figure 1D, Table S1B). At Harvey’s Marsh (a low resource site), total visitation to plants remained constant, irrespective of floral density, but there was a negative relationship between the per-unit resource visitation rates and flower density, again suggesting a resource dilution effect. On the other hand, at Brooklyn (a high resource site), total visitation rates increased with flower density, but there was no effect of floral density on the per-unit resource visitation rates. The total time that all visitors spent on *R. triloba* flowers was significantly greater at Harvey’s Marsh (52.0 min per plant) than at Brooklyn (26.3 min per plants, *F*_1,24_ = 4.84, *p* = 0.03; Table S1B).

### 3.2. Seed Removal

There was a positive relationship between flower treatment and seeds produced (*V. stricta* *F*_2,24_= 18.00, *p* < 0.01, *R*^2^ = 0.44; *R. triloba* *F*_2,24_ = 28.15, *p* < 0.01, *R*^2^ = 0.65), therefore individuals belonging to any one of the three flower treatments (“low”, “medium” and “high” flower treatment) had similar corresponding seed densities (for *R. triloba*: mean “low” seed density = 16.7 ± 3.25 (SE), “medium” seeds = 82.5 ± 16.93, “high” seeds = 197.5 ± 30.61; for *V. stricta*: mean “low” seed densities = 108.4 ± 28.5, “medium” seeds = 207.3 ± 54.47 and “high” seeds = 456.6 ± 65.34). There was also a significant difference in the number of seeds produced for *V. stricta* (*F*_2,24_ = 13.65, *p* < 0.01). *Verbena stricta* plants in Harvey’s Marsh (a low resource site) produced more flowers (mean number of seeds per plant = 340.6 ± 58.14 (SE) than plant at Brooklyn (a high resource site, mean number of seeds per plant = 174.2 ± 46.32). 

**Figure 2:**
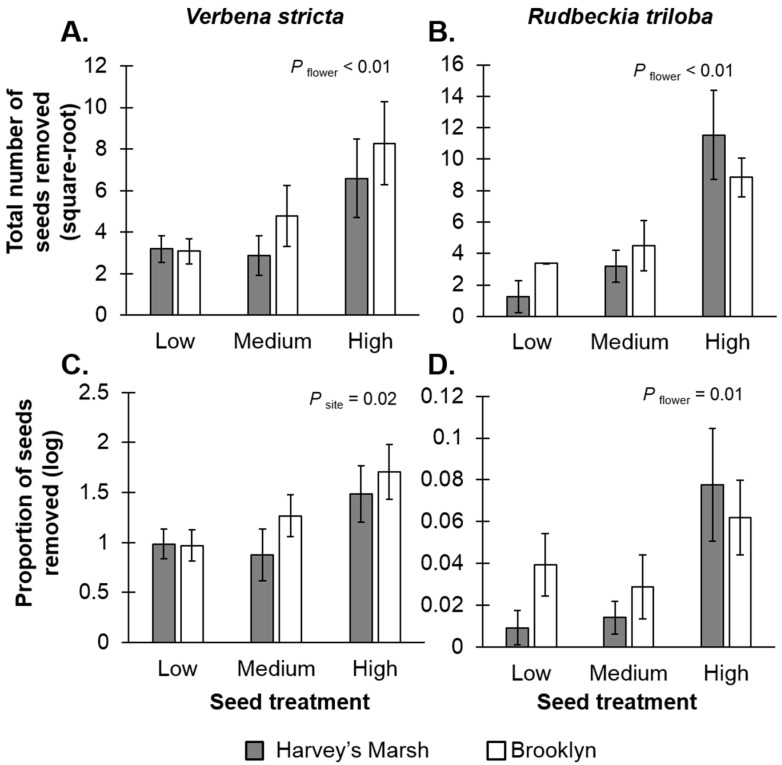
Seed treatment and site comparisons of *Verbena stricta* (left side) and *Rudbeckia triloba* (right side) on the total number of seeds removed (**A**,**B**) and the proportion of seeds removed after 48 h of exposure (**C**,**D**). Grey bars represent rates at Harvey’s Marsh (a low resource density site); white bars represent rates at Brooklyn (a high resource density site). Error bars represent ±1 SE. The total number of seeds removed was square-root transformed, and the proportion of seeds removed was log-transformed.

Seed resource densities affected seed removal rates. For *V. stricta*, there was a positive relationship between seed density and the total number of seeds removed (*F*_2,24_ = 5.69, *p* < 0.01; Figure 2A, Table S1A), suggesting resource concentration effects, but no relationship with seed-resource density and the per-unit resource removal rates (*F*_2,24_ = 0.05, *p* = 0.94; Figure 2C, Table S1A). The per-unit resource removal rates did vary with site (*F*_1,24_ = 5.78, *p* = 0.02); there were higher seed predation rates at Brooklyn, a high resource site (mean removal rates 28%) compared to Harvey’s Marsh, a low resource site (mean removal rates = 7.5%). For *R. triloba*, there were positive relationships with resource densities and removal rates (total seeds removed: *F*_2,24_ = 12.74, *p* < 0.01, Figure 2B; per-unit removal rate: *F*_2,24_ = 4.66, *p* = 0.01, Figure 2D, Table S1B) again, suggesting resource concentration effects. Site had no effect on removal rates (*F*_1,24_ < 0.01, *p* = 0.92) nor was there an interaction between site and seed treatment (*F*_2,24_ = 1.23, *p* = 0.31). 

### 3.3. Fitness Consequences of Pollination and Seed Predation for Plants

For *R. triloba*, insect pollination was important for seed set because experimental (unbagged) plants produced significantly more seeds (mean = 101.75 seeds) than the control (bagged) plants (mean = 50.12 seeds, *F*_1,43_ = 3.96, *p* = 0.05). For *V. stricta*, unbagged experimental plants produced a similar number of seeds (mean = 257.4 seeds) as control plants (mean = 277.33, *F*_1,46_ = 0.08, *p* = 0.76), indicating that insect pollination was not important for *V. stricta* seed set.

**Figure 3:**
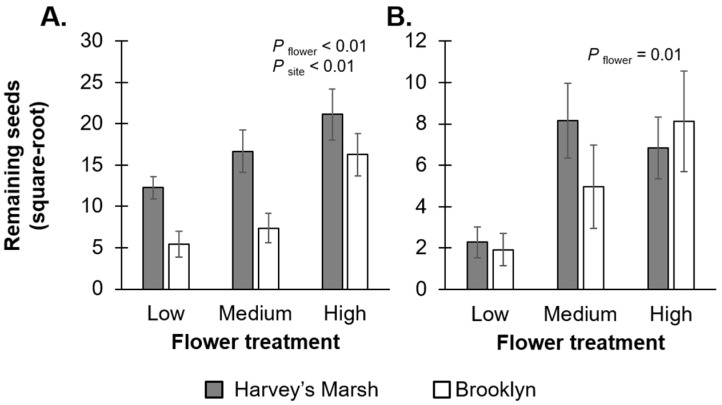
Flower treatment and site comparisons on the overall number of seeds remaining after the pollination and seed predation experiments on *Verbena stricta* (**A**) and *Rudbeckia triloba* (**B**). Grey bars represent rates at Harvey’s Marsh (a low resource density site); white bars represent rates at Brooklyn (a high resource density site). Error bars represent ±1 SE. Remaining seed numbers were square-root transformed.

At the end of both the pollination and seed predation experiments, there were positive relationships between the initial number of flowers on the plant and the final number of seeds remaining for *V. stricta* (F_2,24_ = 10.29, *p* < 0.01; Figure 3A, Table S1A) and *R. triloba* (F_2,24_ = 5.25, *p* = 0.01; Figure 3B, Table S1B). For *V. stricta*, the final number of seeds was also strongly affected by site (*F*_1,24_ = 14.85, *p* < 0.01); Brooklyn had more seeds left over after the pollination and seed removal experiments. On the other hand, for *R. triloba*, there was no difference in the final number of seeds between the two sites (*F*_1,24_ = 0.26, *p* = 0.60).

## 4. Discussion

In this prairie system, resource density influenced the visitation and seed removal rates of two common forbs, but the relationships varied with consumer type. In general, we found negative relationships between floral resource density and the visitation rates of potential pollinators for both plant species, suggesting resource dilution effects. That is, as resources became more abundant locally, plants received relatively fewer visits per flower. In contrast, we found a positive relationship between resource density and seed removal rates by seed predators in support of the resource concentration effect. We also found that pollination and seed removal rates were site dependent. Plants in Brooklyn (a high resource density site) received fewer visits to flowers, but suffered greater seed removal rates. These findings indicate that pollinators and seed predators may respond differently to variation in resource density and that future studies should consider life history traits and foraging behavior when investigating resource concentration effects.

### 4.1. Possible Mechanisms of Density Effects on Pollination and Seed Predation

We found different relationships between resource density and foraging rates for pollinators and seed predators: resource dilution effects for visitation rates, but resource concentration effects for seed removal. One possibility for the different relationships between resource density and foraging rates could be due to the different life history traits of the consumers. Work by others (see [33,34]) has found similar resource dilution effects for wild (solitary) bees, but resource concentration effects for managed (social) honey bees. Social insects, such as honey bees and ants, use recruitment-based foraging to inform other colony members of available resources. If there is a greater density of resource available, conveying information to other members of the colony might be advantageous for the colony, thus leading to a greater number of individuals concentrating at the resource location. On the other hand, solitary insects, such as some wild bees, may want to minimize competition for resources and/or avoid predation risks while foraging and, therefore, might forage in less desirable areas for food [13]. Dilution effects may also occur due to saturation, where in resource-dense patches, there are not enough resources for foragers to distribute their foraging efforts compared to resource-sparse patches [23]. In our study, because the total visits to plants remains constant (or increased) across flower density treatments, the negative relationships between resource density and the per-unit resource visitation rates on plants is likely due to saturation effects for *V. stricta* (Figure 1A,C) and *R. triloba* (Figure 1B,D).

### 4.2. Context-Dependency in Pollination and Seed Removal Rates

Pollination rates varied with site where plants at Harvey’s Marsh (a low resource density site) received more visits than Brooklyn (a high resource density site). One reason for the site-level differences could be due to the difference in the relative quantity of naturally-occurring floral resources between sites. The availability of naturally-occurring resources at Harvey’s Marsh was low; therefore, as we predicted, pollinators may have been attracted to the newly-placed experimental plants at the site and visited plants from all resource-density treatments equally (*i.e.*, visitors were not selective about which plants they visited). On the other hand, Brooklyn had greater abundance of naturally-occurring floral resources; therefore, our experimental plants might not have been considered attractive to the pollinators relative to the other plant species within the field. Pollinators that did visit the experimental plants may have been more selective, especially with *R. triloba*, where pollinators visited plants with greater floral resources compared to plants with fewer flowers.

Site also affected seed removal rates, but only for *V. stricta* seeds. Between-site differences could be due to the overall differences in ant abundances and seed preference. In a previous study [29], Brooklyn had greater ant abundances (2612 individuals, mainly *Lasius neoniger*) compared to Harvey’s Marsh (739 individuals) and could explain why there was greater removal rates in Brooklyn compared to Harvey’s Marsh. It is unclear, however, why site level differences were observed for *V. stricta* and not *R. triloba*. One possibility could be due to ant preference for *V. stricta* seeds compared to *R. triloba* (*V. stricta* mean removal rate = 19.2% *vs. R. triloba* mean removal rate = 9.4%: *t* = 1.95, *df* = 57, *p* = 0.05). If ants preferred *V. stricta*, then demand for these resources would be greater at the site with more ants (Brooklyn). Conducting feeding trials with ants would help elucidate whether seed preferences contributed to differences between sites, and conducting similar studies at more sites would be helpful to determine the generality of the results.

### 4.3. Potential Fitness Consequences of Visitation and Seed Predation for Plants

Plants may face trade-offs between producing many flowers to attract pollinators, but not too many to attract seed predators, resulting in balancing selection towards an optimal phenotype [24]. In our study, we found that plants with a low number of flowers had greater visitation rates and also lower seed removal rates, favoring a phenotype with fewer flowers. Producing fewer flowers might be an optimal strategy for various reasons. First, if plants produce fewer flowers with limited resources, the resulting seeds may be larger in size or quality, which could increase the likelihood of germination or survival [35]. Second, our study plants were perennials, so investing less energy towards reproduction and more towards growth may be the optimal strategy for lifetime fitness and survival [36].

While producing fewer flowers might seem like an optimal strategy to increase visitation rates and lower seed removal rates respectively, the total (net) seeds remaining at the end of the experiment (after pollination and seed predation) was greater in the high floral resource treatment than the low floral resource treatment. When considering the total number of seeds remaining (rather than pollination and seed predation rates), this suggests that the optimal strategy for plants is the opposite; to produce many flowers. One caveat, however, is that this study was conducted for a short period where the duration of the pollination and seed removal experiments varied (one week for the pollination experiment and 48 h for the seed removal experiment). While the short time frame in our study allowed us to quantify visitation and seed removal rates when resources are the most “attractive”, it is possible that our snapshot of fitness was not representative of the whole season or lifetime fitness for plants. Furthermore, we only focused on post-dispersal seed removal rates and did not estimate pre-dispersal seed removal rates, which could increase the effects of seed predators on plants. A longer study examining the interaction between pollinators and seed predators (both pre- and post-dispersal) would allow us to better examine the evolutionary implications of pollinators and seed predators on the phenotype of plants.

## 5. Conclusions

We found resource concentration effects with seed predators and resource dilution effects with pollinators. These opposing relationships might be due to different foraging behaviors of the consumers where seed predators use a recruitment-based strategy, whereas pollinators were largely solitary foragers and saturation effects are likely. Furthermore, foraging rates were context-dependent, suggesting that site-level differences in the relative availability of resources and consumer abundances might influence foraging behavior.

## Supplementary Materials

The following are available online at www.mdpi.com/2075-4450/7/2/23/s1, Figure S1: Experimental layout of plants within each site, Table S1: ANOVA tables for floral density and site effects on foraging rates.
