# Supplementary material for: Contrasting Foraging Patterns: Testing Resource-Concentration and Dilution Effects with Pollinators and Seed Predators
Source: Insects. 2016 Jun 3;7(2):23. doi: 10.3390/insects7020023 (PMC4931435; doi:10.3390/insects7020023)
Supplement: Supplementary file 1 [file insects-07-00023-s001.pdf]

insects

MDPI

Supplementary Materials: Contrasting Foraging Patterns: Testing Resource-Concentration and Dilution Effects with Pollinators and Seed Predators

Alexandria Wenninger, Tania N. Kim, Brian J. Spiesman and Claudio Gratton

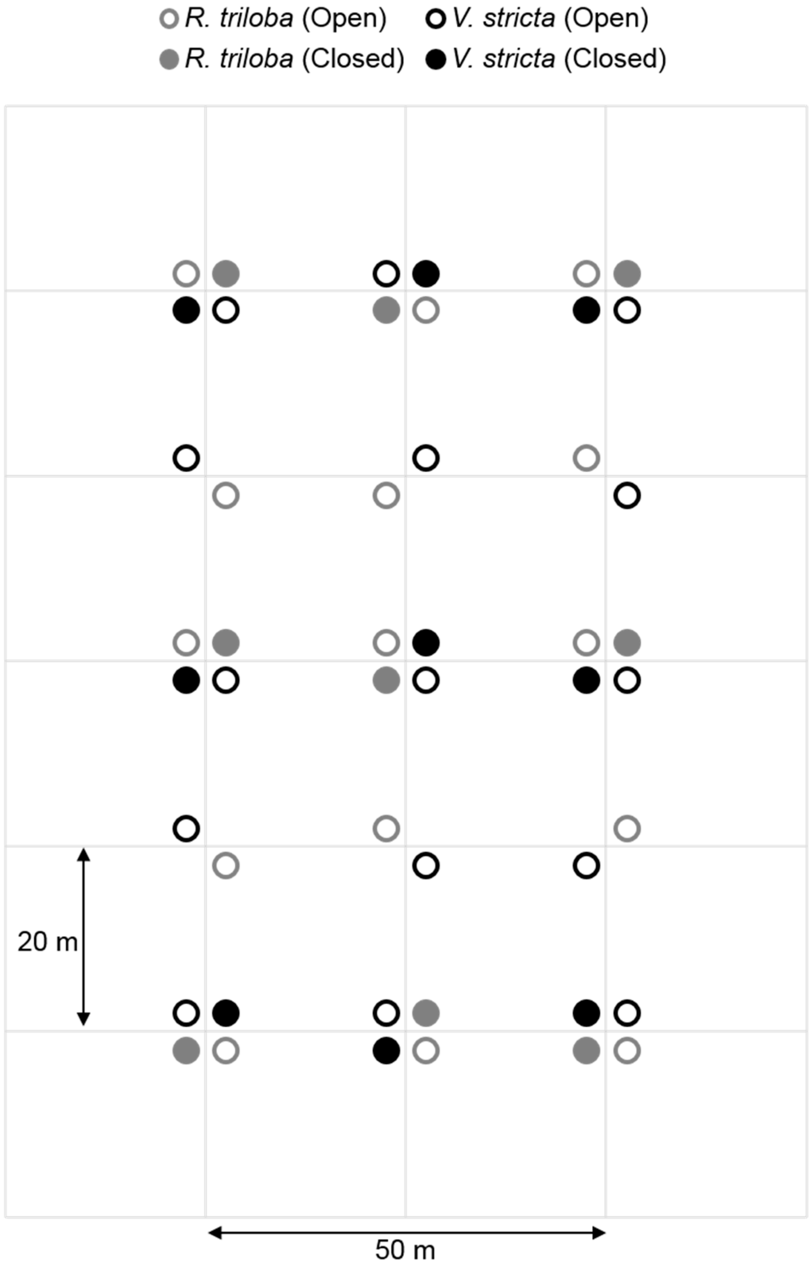

Figure S1. Experimental layout of plants within each site. Points represent a single potted plant (either *Rudbeckia triloba* or *Verbena stricta*). Plants at each station were separated by >1.5 m from each other; stations were separated by >20 m. "Open" are plants with unbagged flowers (exposed to pollination, n = 30 individuals); "Closed" are plants with bagged flowers (preventing pollination, n = 18 individuals). Total number of plants per site = 48 individuals.

Table S1. ANOVA tables for floral resource density and site, effects on total number of visits (square-root transformed), visits per flower, total visitation time (log-transformed), number of seeds produced (square-root transformed), total seeds removed (square-root transformed), proportion of seeds removed (log-transformed), and final number of seeds remaining at the end of the experiment (square-root transformed). Table S1Afor *Verbena stricta* and Table S1B for *Rudbeckia triloba*.

	Predictor Variables					
**(A)** ***Verbena Stricta***	**Floral Density**		**Site**		**Floral Density × Site**	
**Response Variables**	$F_{2,24}$	p	$F_{1,24}$	p	$F_{2,24}$	p
Total visits	2.67	0.08	7.77	**0.01**	0.05	0.94
Visits per flower	5.42	**0.01**	8.02	**<0.01**	1.08	0.35
Total visitation time	0.66	0.52	2.61	0.11	1.45	0.25
Seeds produced	18.00	**<0.01**	13.65	**<0.01**	0.36	0.69
Total seeds removed	5.69	**<0.01**	1.03	0.31	0.27	0.76
Proportion of seeds removed	0.05	0.94	5.78	**0.02**	0.49	0.61
Final seeds number	10.29	**<0.01**	14.85	**<0.01**	0.47	0.62

	Predictor Variables					
**(B)** ***Rudbeckia Triloba***	**Floral Density**		**Site**		**Floral Density × Site**	
**Response Variables**	$F_{2,24}$	p	$F_{1,24}$	p	$F_{2,24}$	p
Total visits	3.64	**0.04**	<0.01	0.97	3.65	**0.04**
Visits per flower	0.21	0.80	0.01	0.91	6.67	**<0.01**
Total visitation time	0.16	0.84	4.84	**0.03**	0.06	0.93
Seeds produced	28.15	**<0.01**	0.39	0.53	0.64	0.53
Total seeds removed	12.74	**<0.01**	<0.01	0.92	1.23	0.31
Proportion of seeds removed	4.66	**0.01**	1.85	0.18	0.74	0.48
Final seeds number	5.25	**0.01**	0.26	0.60	0.92	0.40

© 2016 by the authors; licensee MDPI, Basel, Switzerland. This article is an open access article distributed under the terms and conditions of the Creative Commons by Attribution (CC-BY) license (http://creativecommons.org/licenses/by/4.0/).
